# Rare solitary neoplasm of the costa in an adult: a case report

**DOI:** 10.1186/1477-7819-12-297

**Published:** 2014-09-25

**Authors:** Cheng Shen, Yasha Liang, Huan Xu, Guowei Che

**Affiliations:** Department of Thoracic Surgery, West-China Hospital, Sichuan University, Guo xue xiang, No.37, Chengdu, 610041 China; Department of General Practice, West-China Hospital, Sichuan University, Guo xue xiang, No.37, Chengdu, 610041 China; Department of Pathology, West-China Hospital, Sichuan University, Guo xue xiang, No.37, Chengdu, 610041 China

**Keywords:** Solitary plasmacytoma, Surgery

## Abstract

Regional solitary plasmacytoma of the costa is a rare disease and is characterized by only one or two isolated bone lesions. We report the case of a solitary plasmacytoma of the bone of the right chest wall in an adult. The patient underwent complete *enbloc* resection of the chest wall including ribs, muscle and parietal pleura. The patient is asymptomatic without any recurrence after two months of follow-up.

## Background

Primary malignant tumors of the bony chest wall are rare and localized solitary plasmacytoma of bone (SPB), characterized by only one or two isolated bone lesions with no evidence of disease dissemination, is a rare disease that accounts for about 5% of malignant plasma cell tumors [1]. We report the case of SPB in the right chest wall.

## Case presentation

A 54-year-old the Han Nationality male was admitted to our hospital presenting with continuous pain in the right chest for the past 3 months. The pain was sudden in onset, non-radiating and increased on coughing. He was a nonsmoker and had no exposure to any known toxic environmental fumes or dusts.

**Figure 1:**
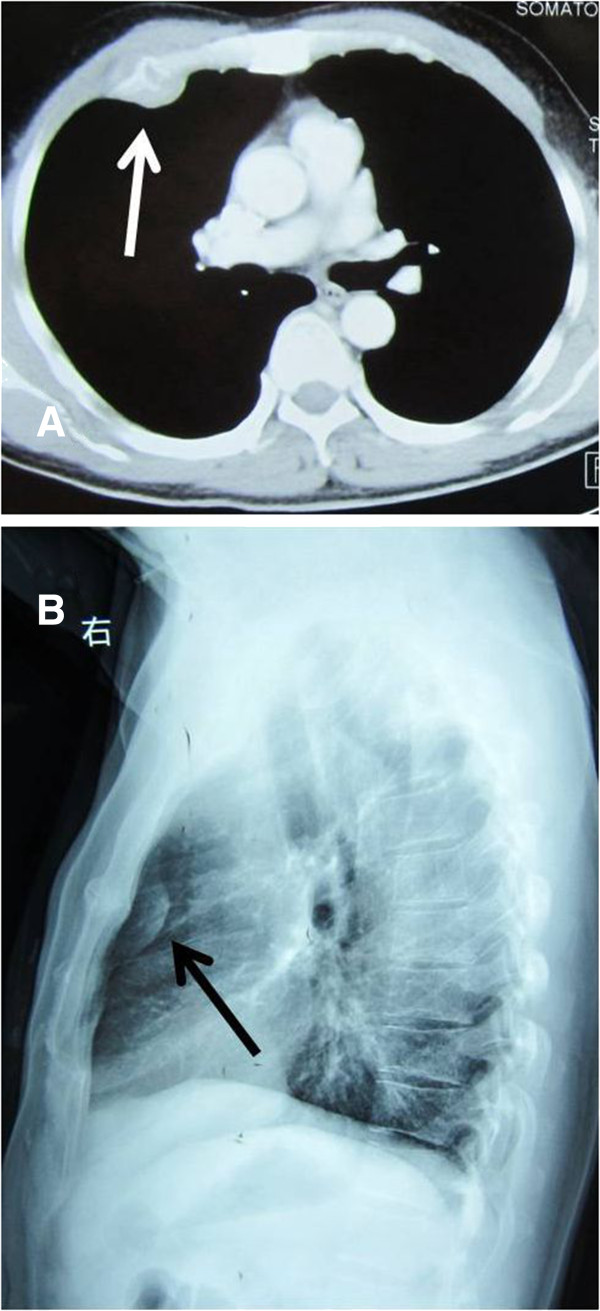
**Chest X-ray and CT showed a mass on the right side of the chest wall. A** and **B**: Chest X-ray and contrast-enhanced CT revealed a well-defined soft tissue mass (arrowhead) on right side abutting the chest wall, with invasion into the third rib.

**Figure 2:**
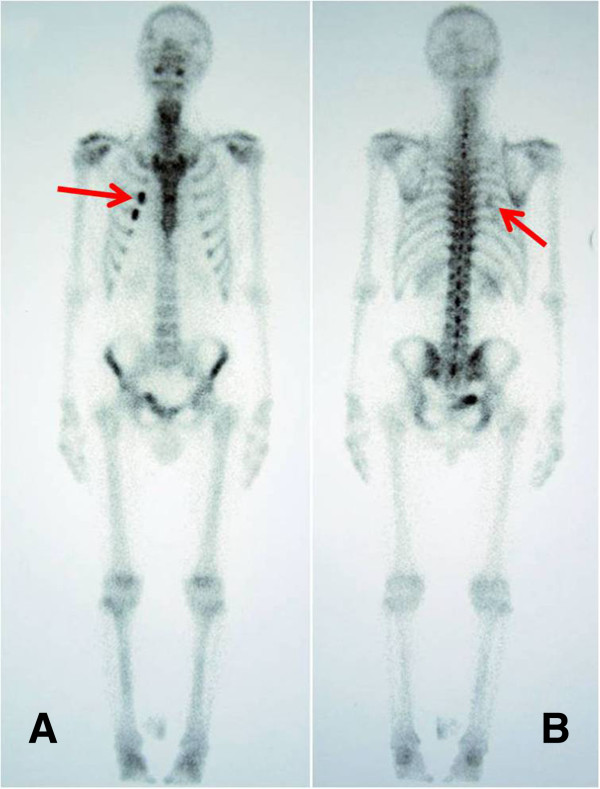
**Bone scan of the patient.** Abnormally increased uptake of radioisotope in the right third and fourth ribs in a bone scan. **A**: anterior to posterior view. **B**: posterior to anterior view. Arrows indicate the tumor.

A physical examination showed an elliptical swelling on the right side of the chest over the third rib, about 3 × 4 cm in size, firm and tender. His chest X-ray showed a pulmonary opacity with well-defined medial and lateral margins merging with chest wall on right side (Figure 1B). A computed tomography scan (CT) revealed a well-defined hypodense soft tissue mass, about 3.2 × 4.1 cm in size, on right side of the chest wall with invasion into the third rib (Figure 1A). An additional bone scan found an abnormal increase in the uptake of radioisotope in the third and fourth ribs (Figure 2A,B). Upon performing abone marrow biopsy no excess of plasma cells were found. A urine analysis for Bence Jones proteins was negative.

**Figure 3:**
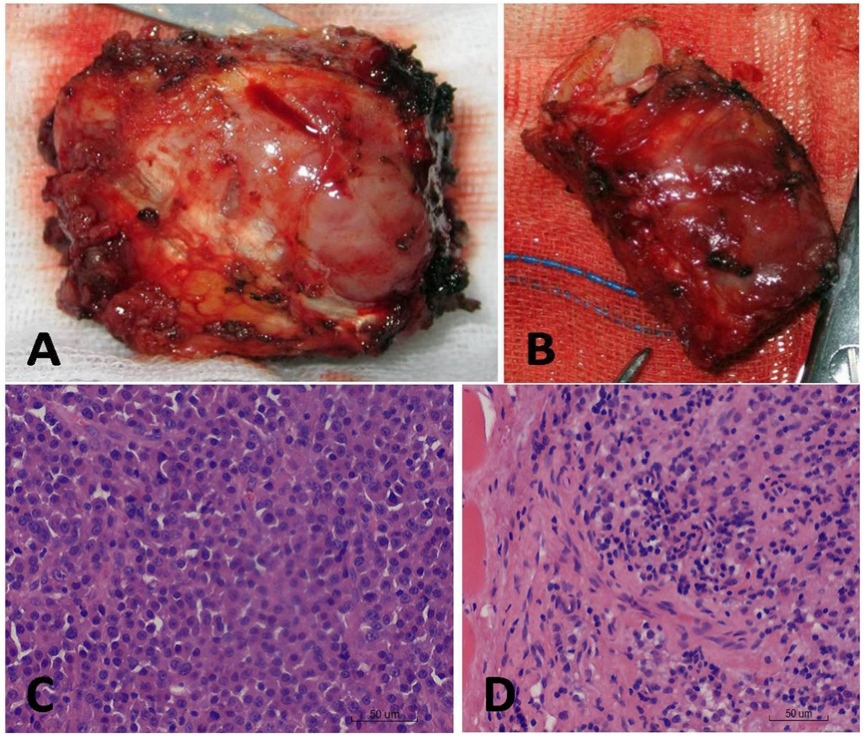
**Macroscopic view of the specimen and histological features. A** and **B**: Macroscopic view of the third and fourth excised ribs with the tumor. **C** and **D**: Histological examination showed many neoplastic plasma cells with the tumor. (hemotoxin and eyolin, ×400 magnification).

As diagnosis was not established through imaging, surgery was scheduled. Under general anesthesia with selective intubation, the patient was subjected to the right posterolateral thoracotomy through the fourth intercostal space which revealed a well-restricted tumor of 4.5 × 5 cm in size arising from the third and fourth ribs anteriorly (Figure 3A, B). *Enbloc* resection of third and fourth ribs, along with the parietal pleura and muscles, was performed with a 5 cm tumor margin and primary closure was done. Microscopically, the lesion was composed of many plasma cells as a thick sheet with scant intercellular stroma. The plasma cells had extensive basophilic cytoplasms, round eccentric nuclei with a cartwheel appearance and perinuclear halos (Figure 3C, D). Immunohistochemical staining revealed that the plasma cells were negative for CD20, cyclin D1, Igκ, IgG and IgM. The plasma cells were positive for D138, PC, mum1 and Igλ. The positive rate of Ki67 was 60%. The histology and immunohistochemical staining results supported the diagnosis of plasmacytoma. There was no serum evidence of M-protein. The patient was doing well without evidence of tumor recurrence or secondary neoplasms at two months following his initial diagnosis.

## Discussion

Plasmacytoma involving the chest wall is a rare primary tumor [2] classified as multiple myeloma (MM), solitary plasmacytoma, extramedullary plasmacytoma or plasma cell leukemia. More than 95% of these tumors are MMs [3]. A solitary plasmacytoma in a rib usually shows destruction of the bone cortex with extension into the surrounding soft tissues. Plasmacytoma may be primary or secondary to the disseminated multiple myeloma and may arise from the osseous or non-osseous sites.

The ratio of male to female patientsis approximately 1.3:1 and the average age on presentation is 59.5 years with a range from 39 to 77 years [3]. The most common symptoms are pain and spinal nerve compression. In our case, the patient (who was a 54-year-old male) had a symptom of constant pain in the chest on the right side with exacerbation upon coughing. With regard to radiological evaluation, chest radiographs are the most commonly performed imaging analysis to evaluate lumps, but may not always provide a conclusive diagnosis. In such cases, a spiral CT is the next tool of choice. Plasmacytoma is typically seen on a CT scan as well-defined, ‘punched-out’ lytic lesions with associated extrapleural soft tissue mass. In advanced plasmacytoma, there is often marked erosion, expansion and destruction of bone cortex, sometimes with thick ridging around the periphery, creating a ‘soap bubble’ appearance [4]. There is also a word in our case. The patient’schest X-ray with lateral projection clearly showed a pulmonary lump and his contrast-enhanced CT revealed a well-defined hypodense soft tissue mass on the right side of the chest wall with invasion into the third rib. The differential diagnosis of costal plasmacytoma includes chondrosarcoma, lymphoma, metastases, osteosarcoma, fibrosarcoma, histiocytoma, chondroma, lipoma and bone infarction [5].

The purpose of the treatment of SPB is to prevent transformation into MM and recurrence. In the past, radiation therapy was used as the primary treatment for SPB because this tumor is sensitive to radiotherapy. Although there is no established relationship between dosage and response; 40 to 50 Gy radiation in 20 to 25 fractions has usually been recommended [6]. Mendenhall *et al.* reported a 6% local failure rate in patients with solitary plasmacytoma treated with doses of 40 Gy or above, in contrast to a 31% failure rate for doses below 40 Gy [7]. Aviles *et al.* discerned that most patients treated with adequate radiation therapy alone will develop MM within the first three years after diagnosis and treatment [8]. When performing surgical resection, the possibility of structural instability and nerve injury should be considered for spinal lesions. For other lesions, variable surgical strategies can be applied according to the tumor size, extent, patient’s general condition or surgeon’s experience [6]. One group reported that the primary methods for treating solitary plasmacytoma were surgery plus radiation therapy in 95 cases and surgery alone in 15 cases. They showed the lowest incidence of progressive diseases in patients with solitary plasmacytoma who were treated with surgery plus an adequate dose of radiation therapy [9]. In our case, we had referred the patient firstly for a wide excision of a solitary rib tumor containing normal tissue without preoperative confirmation of diagnosis. The histopathologic diagnosis of SPB was confirmed, and a treatment plan with doses of 40 Gy radiation in 25 fractions was planned for protection against tumor recurrence.

## Conclusions

In conclusion, there are very few reports in the literature depicting the natural history of SPB in an adult in regard to its presentation, pathology and treatment [10]. Our case may help recognition of this rare disease in the thoracic surgery field, thus avoiding misdiagnosis and inadequate treatment.

## Consent

Written informed consent was obtained from the patient for publication of this case report and any accompanying images. A copy of the written consent is available for review by the Editor-in-Chief of this journal.
